# Effect of message framing on support for a sugar-sweetened beverage tax in Australia: a cross-sectional survey analysis

**DOI:** 10.1093/heapro/daad193

**Published:** 2024-01-11

**Authors:** Katherine Cullerton, Anastassia Demeshko, Michael Waller

**Affiliations:** School of Public Health, The University of Queensland, 266 Herston Rd, Herston, Queensland 4006, Australia; School of Public Health, The University of Queensland, 266 Herston Rd, Herston, Queensland 4006, Australia; School of Public Health, The University of Queensland, 266 Herston Rd, Herston, Queensland 4006, Australia

**Keywords:** health policy, message framing, health communication, sugar tax, advocacy

## Abstract

Sugar-sweetened beverage (SSB) taxes are present in many countries with evidence that they are effective in decreasing purchases of SSBs. However, in Australia where SSB consumption per capita is high, and calls for an SSB tax are frequent, there is no SSB tax and policymakers have stated their lack of support for such a tax. We examined whether political party voting preference and sociodemographic factors affect individuals’ support for an SSB tax, and whether message framing affects this support. A nationally representative sample of 1519 Australian adults was recruited for an online experimental survey. Three persuasive frames and one control frame were randomly provided to participants and measures of agreement towards an SSB tax were assessed. Sociodemographic factors and political party preference were also captured. Message framing had minimal effect on the level of support for the tax. However, participants who received the ‘supportive of food and drink companies frame’ showed the highest positive feelings towards the tax, and participants in rural areas had higher levels of support for an SSB tax when receiving the ‘protecting teenagers’ frame. Participants who voted for conservative (right-leaning) parties and for Labour (a centre-left party) had similar levels of support towards the tax, which was considerably lower than Greens voters. Undecided voters had the lowest levels of support for the tax, and the frames had limited impact on them. These findings highlight the potential role of message framing in shaping public support for an SSB tax in Australia, particularly in the context of voting preference and sociodemographic factors.

**Contribution to Health Promotion**

- Message framing is a popular strategy used by health promotion advocates.
- However, we found it has a minimal effect on the level of support for a sugar-sweetened beverage tax.
- The ‘supportive of food and drink companies’ frame received the highest positive feelings.
- The ‘protecting teenagers’ frame particularly resonated with participants from rural areas.
- Undecided voters had the lowest support for the tax and frames had minimal impact with them.

## BACKGROUND

Making healthy dietary choices is an increasing challenge in our current food environment. This is reflected by the rising rates of obesity and non-communicable disease (NCD), with one third of all adults living with overweight or obesity and 41 million deaths attributed to NCDs worldwide (World Health Organization., 2022b). While many factors have contributed to the rising rates of NCDs, consuming high amounts of sugar is associated with an increased risk of developing NCDs (Chazelas *et al.*, 2019; Malik *et al.*, 2019). Notably, a consistent association has been found between high consumption of sugar-sweetened beverages (SSBs) such as carbonated and non-carbonated soft drinks, sports drinks and juices and developing obesity (Morenga *et al.*, 2013; Bleich and Vercammen, 2018).

One proposed solution to counter high sugar consumption from SSBs is an SSB tax (World Health Organization, 2016). Utilizing taxes that apply to SSBs is a popular and effective strategy to reduce consumption with such taxes currently in place in 85 countries (World Health Organization., 2022a). SSB taxes have been associated with lower demands and purchasing of SSBs in locations such as Mexico, UK and parts of the USA (Colchero *et al.*, 2016; Public Health England, 2020; World Bank, 2020). However, in Australia where SSB consumption per capita is high, and calls for an SSB tax have been frequent, there is no SSB tax and some policymakers have publicly stated that they do not support such a tax (Teng *et al.*, 2019; Cancer Council, 2021).

Support for an SSB tax from the Australian public has been mixed with support ranging from 33% to 60% (Cullerton *et al.*, 2021; Pettigrew *et al.*, 2023). This level of support is lower than other proposed public health nutrition policies, such as banning highly processed food advertising to children (57–86%), and providing health warning labels on food (71–89%) (Cullerton *et al.*, 2021). Generally, public acceptability of policy-level interventions targeting behavioural change is lowest for policies considered intrusive, like an SSB tax, as the public often sees fiscal health policies as a threat to their decision-making ability (Diepeveen *et al.*, 2013). These low levels of support are concerning for advocates as public support for a proposed policy is a factor in increasing the likelihood of policy change (Cullerton *et al.*, 2018).

Other information relevant to policymakers when they are considering policy change is which political party supporters of a proposed policy align with (Cullerton *et al.*, 2021). Policymakers and politicians want to know these details as it provides them with an opportunity to appeal to their political base while simultaneously appealing to swing, or undecided, voters (Druckman and Jacobs, 2006). In Australia, where voting is compulsory, a significant proportion (approximately 30%) of the population are considered swing or undecided voters (Cameron and McAllister, 2019). However, few studies have examined the political leanings of the supporters (or dissenters) of public health policies (Cullerton *et al.*, 2022). This lack of nuanced data may impact the likelihood of policymakers supporting the introduction of an SSB tax and is an area that requires further investigation.

The use of message framing, that is, selecting some aspects of perceived reality and making them more salient in a communicating text (Entman, 1993), is a popular strategy used by advocates to increase policy support from policymakers and the general public (Cullerton *et al.*, 2018). The use of message framing has been shown to increase support for health policy interventions, particularly for those who do not have a strong opinion on a policy issue (Roh and Niederdeppe, 2016; Gollust *et al.*, 2017). However, for a message frame to be effective it must be *available* (e.g. audience understands and relates to the concept), *accessible* (e.g. audience has regular or recent exposure to the concept) and *applicable* to the audience (Chong and Druckman, 2007). Factors that increase applicability include the quality or logic of the argument, source credibility and message relevance (Bullock and Shulman, 2021).

Political ideology can also play an important role in influencing responses to message framing and consequently, levels of public health policy support (Lee and Kim, 2016; Gollust *et al.*, 2017). In the USA, exposing respondents to messages about the role of social determinants in health outcomes (e.g. those living in low-income neighbourhoods have higher rates of diabetes) only increased public health policy support among Democrats relative to the control; while Republicans/other parties had no change (Gollust *et al.*, 2009). This, alongside the influence of other demographic characteristics, such as gender and education level, reinforces the challenges in communicating about public health policy.

Numerous experimental framing studies on nutrition-related policies have been conducted internationally (Barry *et al.*, 2013; Gollust *et al.*, 2013, 2017; Lee and Kim, 2016). While it is important to learn from overseas populations about effective and ineffective message frames, communication is contextual. This means a message frame that works in one country may not work in another (Kreuter and McClure, 2004). Message frames may not resonate with different population sub-groups within a country, for example, those in rural locations or with different political ideologies (Slater, 1995). To further understand the role of context in message framing, we aimed to examine the impact of different frames on support for an SSB tax in Australia, considering the role that different attributes such as age, gender and location, as well as political party voting preferences play in impacting levels of support. Understanding the role of these contextual factors in the support of health policy may provide insightful data for advocates or politicians hoping to reach additional voters.

### Objectives

Our objective was to examine whether political party voting preference and sociodemographic factors affect individuals’ support for an SSB tax, and to identify whether message framing affects this support. Therefore, we aimed to investigate: (i) the agreement-with, perceived effectiveness, and feeling-towards the introduction of a 20% SSB tax in Australia; (ii) how voting preference and sociodemographic factors influence these perspectives and (iii) how message framing affects the level of support of the SSB, and whether any factors moderate an increase in support. Specifically, we wanted to understand whether support for SSB interacted with political party voting preference, geographic area, gender and age.

## METHODS

### Frames utilized

The frames used in this study were developed using previous findings regarding persuasive frames (Reynolds *et al.*, 2020) as well as data from street intercept interviews designed to gain insight into the public’s thoughts and beliefs about different nutrition policies, including an SSB tax (Cullerton *et al.*, 2022). Using the terms and concepts that people readily use when discussing an issue as the basis for a frame should influence the concepts of accessibility and availability as suggested in framing theory (Chong and Druckman, 2007). This also aligns with the theory of concept accessibility, that is, people often base their opinions on accessible concepts without conscious deliberation (Zaller and Feldman, 1992). With these two theories in mind, we developed four messages to test different frames (Table 1). This included a control message (Block 1), a message frame pertaining to protecting teenagers (Block 2), a frame supportive of food and drink companies (Block 3) and an anti-sugary drink companies framed message (Block 4). Each message was developed with the input of two communications professionals and was provided to participants as an online newspaper ‘brief’. The messages were kept brief intentionally to avoid the risk of participant disengagement associated with longer messages (Van Dijk, 2013). To ensure the different frames were not undermined by the use of multiple conflicting terms, the messages remained largely consistent with only minor changes made to represent the different frames. The message frames were pilot tested among 12 participants representing diverse demographics, this resulted in minor refinements to enhance message clarity.

**Table 1:** Message blocks used

Frame	Message statement
Block 1: Control	As a nation we are eating and drinking too much sugar, this is contributing to high rates of obesity. One way to decrease the amount of sugar we consume is to add a 20% tax onto the price of sugary drinks. This has been an effective strategy in other countries around the world to decrease the amount of sugary drinks people consume.
Block 2: Protecting teenagers	As a nation we are eating and drinking too much sugar, this is contributing to high rates of obesity. This is particularly a problem with Australian teenagers. One way to decrease the amount of sugar teenagers consume is to add a 20% tax onto the price of sugary drinks. By increasing the price a little it will help teenagers think twice before purchasing sugary drinks. This has been an effective strategy in other countries around the world to decrease the amount of sugary drinks people consume.
Block 3: Supportive of food and drink companies	As a nation we are eating and drinking too much sugar, this is contributing to high rates of obesity. One way to decrease the amount of sugar we consume is to add a 20% tax onto the price of sugary drinks. This tax will encourage food and drink companies to sell healthier products that do not cost us, the taxpayer, more in health care costs. It will also help companies realize they can make more money from healthy products instead of unhealthy ones. This has been an effective strategy in other countries around the world to decrease the amount of sugary drinks people consume.
Block 4: Anti-sugary drink companies	As a nation we are eating and drinking too much sugar, this is contributing to high rates of obesity. One way to decrease the amount of sugar we consume is to add a 20% tax onto the price of sugary drinks. The main opponents of taxes on sugary drinks are soft drink companies. These companies will say and do almost anything to protect their profits. They spend money on marketing, saying that a tax will do nothing, but we know this has been an effective strategy in other countries around the world to decrease the amount of sugary drinks people consume.

In line with our study objectives and previous research, we hypothesized that:

a) Participants who support left and centre-left parties (hereafter referred to as ‘left-leaning’) are more likely to respond to anti-sugary drink companies messaging (Gollust *et al.*, 2009).
b) Females are more likely to be swayed by messaging which concerns protecting children and teenagers (Miller *et al.*, 2019; Pettigrew *et al.*, 2023).
c) Those living in rural areas are more likely to be receptive to messaging which concerns protecting children and teenagers (Winter, 2000).
d) Older individuals are more likely to be influenced by messaging that relates to supporting food and drink companies (Miller *et al.*, 2019).

### Study design, sampling and data collection

We conducted a cross-sectional survey with quantitative and free-text responses throughout December 2019–February 2020. We used two methods to recruit participants, promoting the study via social media accounts and then an online panel from *Qualtrics*, a market research company, to ensure our sample was nationally representative (Qualtrics, 2022). The sample size calculation was based on a 55% agreement-with the SSB tax in the control group and a 65% agreement level in an alternative group, and 70% power. This required 296 people in each group, as we used four frames the target sample size was 296 × 4 = 1184. Participant recruitment exceeded this target (*n* = 1519).

Four different message frames (Table 1) were randomly assigned to participants ensuring the distribution of key characteristics, including gender, geographical area (metropolitan/rural or regional), education status and age were similar between frames. Data was collected using the *Qualtrics* platform (Qualtrics, 2022). Written informed consent was obtained from all respondents prior to data collection. The (anonymized for review) Medicine Low and Negligible Risk Ethics Committee provided ethical approval for this research #2019001612.

The distribution of political party preference was not adjusted for in the recruitment stage; however, percentages were comparable to those reported in the Australian Electoral Survey (Cameron and McAllister, 2019). Therefore, the unweighted sample data was used in the following analyses.

### Variables of interest

The survey and variables that were used in this study are detailed in Appendix 1, Supplementary File. The outcome variables of agreement-with, perceived effectiveness, and feeling-towards the SSB tax were subdivided as measures of agreement, as existing literature has identified differential intentions associated with each (Fielding *et al.*, 2020).

For the agreement-with question (How much do you agree-with the introduction of a 20% tax on sugary drinks?) the outcome used was strongly agree or agree versus, neither agree nor disagree, disagree or strongly disagree. For the perceived effectiveness question (How effective do you think a 20% sugary drinks tax would be in improving the health of Australians?) the outcome used was somewhat effective or very effective versus, neutral, not effective or really not effective. The feeling-towards the SSB tax question (How do you feel about a 20% sugary drinks tax?) was categorized as very positive or positive versus, neutral, negative or very negative.

Sociodemographic factors included gender, age, highest education level attained, Socio-Economic Indexes for Areas (SEIFA) based on the postcode of participants, and geographical area. Additional sample characteristics included political party preference (Liberal/One Nation/National, Labour, Greens, and Other—including Independent party and undecided individuals), soft drink consumption frequency, frequency of thoughts about a sugary drinks tax and how much people follow what is going on in the government and the news. These variables were previously identified as factors that could be associated with greater support for an SSB tax (Cullerton *et al.*, 2021).

### Inclusion/exclusion criteria for data

Adults who consented to participate and completed the full online survey in greater than 2.5 minutes. To confirm the reliability of the responses, participants were also asked to recollect the survey topic before submitting their responses; irrelevant responses were excluded.

### Data analysis

Logistic regression models were generated to understand the associations between the three outcomes of support for the SSB tax, and the exposures of message frames and various sample characteristics. The adjusted OR (95% CI) of support for each outcome was presented. To explore whether the characteristics of political party preference, age, gender and geographical area play a role in framed messaging, interaction terms between these exposure variables and the outcome measures were conducted. To aid with interpretability, the distribution of respondents that were somewhat or strongly in support of the SSB stratified by message framing blocks was presented. The primary interactions of interest included the variables of political party preference, gender, geographical area and age, as these related back to our specified research hypotheses.

## RESULTS

We recruited 1519 nationally representative adults across Australia to participate in the study (see Table 2).

**Table 2:** Summary characteristics of nationally representative population sample (*n* = 1519)

Characteristics	*n* (%)
**Sex**	
Female	774 (51.0)
Male	736 (48.4)
Other	9 (0.6)
**Age**	
18–24 years old	213 (14.0)
25–40 years old	506 (33.3)
41–64 years old	546 (35.9)
65 and over	254 (16.7)
**Education**	
Some secondary	173 (11.4)
Finished secondary	500 (32.9)
Diploma	347 (22.8)
University (undergraduate)	398 (26.2)
University (postgraduate)	101 (6.7)
**Area**	
Metropolitan	1053 (69.3)
Rural	450 (29.6)
Not sure	16 (1.0)
**Political party affiliation**	
Labour	501 (33.0)
Liberal/National/One Nation/Right	533 (35.1)
Greens	212 (13.9)
Independents/Not sure	273 (18.0)
**At least one child at home**	
Yes	501 (33.0)
No	1018 (67.0)
**Main food shopper in household**	
Yes	1077 (70.9)
No	91 (6.0)
Shared	351 (23.1)

### Personal characteristics and levels of support for the SSB tax

Personal characteristics of participants played a role in level of support for the SSB tax. Logistic regression analyses (Table 3) showed that 18–24-year-olds were most likely to perceive an SSB tax to be effective while those over 65 were more likely to feel positive about an SSB tax. Respondents with a university degree were significantly more likely to support the tax (across all three measures) and postgraduates had even higher odds than undergraduates for all question prompts. Political party voting preferences were also consistently associated with positive agreement, perceived effectiveness and feeling-towards the SSB tax. Greens voters consistently had the highest level of support across all three measures, while conservative voters and Labour party voters had similar levels of support for the tax (Agreement-with tax: Greens 76%, Labour 55%, conservative voters 57%). ‘Other’ [Undecided/swing voters (*n* = 199) or Independent party (*n* = 23)] respondents had the lowest level of support for the tax. Other characteristics associated with higher levels of support included those who were frequent news watchers, those who had lower consumption of SSBs and those who frequently thought about an SSB tax.

**Table 3:** Distribution of respondents that were somewhat or strongly in support of the sugar-sweetened beverage tax (*n* (%)) and the adjusted Odds Ratio (aOR) [95% CI] obtained from logistic regression analyses for each message framing block and demographic

Demographics	Q agreement-with tax		Q think tax will be effective		Q positive feeling-towards tax		Subgroup totals
	*n* (%)	aOR (95% CI)	*n* (%)	aOR (95% CI)	*n* (%)	aOR (95% CI)	*n* (%)
**Block**							
Control (Block 1)	203 (55.2)	Baseline	192 (52.2)	Baseline	130 (35.3)	Baseline	368 (24.2)
Protecting teenagers (Block 2)	214 (55.6)	1.06 (0.78, 1.43)	204 (53.0)	1.03 (0.76, 1.40)	151 (39.2)	1.22 (0.89, 1.67)	385 (25.4)
Supportive of food and drink companies (Block 3)	231 (59.2)	1.20 (0.88, 1.62)	215 (55.1)	1.13 (0.84, 1.53)	168 (43.1)	1.40 (1.03, 1.90)*	390 (25.7)
Anti-sugary drink companies (Block 4)	214 (56.9)	1.07 (0.79, 1.45)	195 (51.9)	0.96 (0.71, 1.30)	160 (42.6)	1.33 (0.97, 1.81)	376 (24.8)
Overall	862 (56.7)	–	806 (53.1)		609 (40.0)		1519 (100)
**Age groups**							
18–24 years	124 (58.2)	Baseline	130 (61.0)	Baseline	77 (36.2)	Baseline	213 (14.0)
25–40 years	298 (58.8)	0.83 (0.58, 1.20)	273 (54.0)	0.59 (0.41, 0.84)*	183 (36.2)	0.78 (0.54, 1.13)	506 (33.3)
41–64 years	293 (53.7)	0.79 (0.55, 1.14)	261 (47.8)	0.51 (0.35, 0.73)**	221 (40.5)	1.08 (0.74, 1.57)	546 (35.9)
65 and over	147 (57.9)	0.94 (0.61, 1.44)	152 (55.9)	0.67 (0.44, 1.03)	128 (50.4)	1.67 (1.08, 2.58)*	254 (16.7)
**Gender**							
Male	392 (53.3)	Baseline	378 (51.4)	Baseline	283 (38.5)	Baseline	736 (48.4)
Female	464 (59.9)	1.20 (0.96, 1.50)	422 (54.5)	1.01 (0.81, 1.25)	322 (41.6)	1.13 (0.90, 1.41)	774 (51.0)
Other	6 (66.7)	1.16 (0.26, 5.22)	6 (66.7)	1.33 (0.30, 5.92)	4 (44.4)	1.00 (0.24, 4.16)	9 (0.6)
**Education**							
Some secondary	76 (43.9)	Baseline	74 (42.8)	Baseline	54 (31.2)	Baseline	173 (11.4)
Finished secondary	246 (49.2)	1.04 (0.73, 1.50)	233 (46.6)	1.01 (0.70, 1.45)	162 (32.4)	0.99 (0.67, 1.47)	500 (32.9)
Diploma	179 (51.6)	1.19 (0.81, 1.76)	171 (49.3)	1.25 (0.84, 1.84)	130 (37.5)	1.28 (0.85, 1.93)	347 (22.8)
University undergraduate	284 (71.4)	2.42 (1.62, 3.63)**	256 (64.3)	2.09 (1.40, 3.12)**	299 (50.0)	2.12 (1.40, 3.22)**	398 (26.2)
University postgraduate	77 (76.2)	2.69 (1.49, 4.87)**	72 (71.3)	2.71 (1.52, 4.81)**	64 (63.4)	3.04 (1.72, 5.37)**	101 (6.7)
**Area**							
Metropolitan area	613 (58.2)	Baseline	559 (53.1)	Baseline	432 (41.0)	Baseline	1053 (69.3)
Rural area	241 (53.6)	1.12 (0.86, 1.45)	238 (52.9)	1.21 (0.94, 1.57)	173 (38.4)	1.02 (0.78, 1.34)	450 (29.6)
Not sure	8 (50.0)	1.36 (0.46, 4.06)	9 (47.4)	1.69 (0.56, 5.15)	4 (25.0)	0.97 (0.29, 3.28)	16 (1.0)
**Political party**							
Greens	161 (75.9)	Baseline	154 (72.6)	Baseline	123 (58.0)	Baseline	212 (13.9)
Labour	275 (54.9)	0.48 (0.33, 0.70)**	255 (50.9)	0.47 (0.32, 0.68)**	198 (39.5)	0.48 (0.34, 0.68)**	501 (33.0)
Other^b^	124 (45.4)	0.35 (0.23, 0.53)**	119 (43.6)	0.38 (0.25, 0.57)**	77 (28.2)	0.33 (0.22, 0.49)**	273 (18.0)
Liberal/National/One Nation	302 (56.7)	0.50 (0.34, 0.73)**	278 (52.2)	0.48 (0.33, 0.70)**	211 (39.6)	0.45 (0.31, 0.64)**	533 (35.1)
**SEIFA** ^a^							
Mean (SD): 3.32 (1.46)	–	1.08 (0.99, 1.17)	–	1.00 (0.92, 1.09)	–	0.97 (0.90, 1.06)	–
**Frequency of watching news**							
Hardly at all	68 (39.8)	Baseline	60 (35.1)	Baseline	41 (24.0)	Baseline	171
Only now and then	152 (51.0)	1.50 (1.00, 2.24)*	141 (47.3)	1.68 (1.11, 2.52)*	94 (31.5)	1.37 (0.88, 2.15)	298
Some of the time	323 (58.8)	1.94 (1.33, 2.82)**	302 (55.0)	2.20 (1.50, 3.21)**	222 (40.4)	1.89 (1.25, 2.85)*	549
Most of the time	319 (63.7)	2.25 (1.52, 3.34)**	303 (60.5)	2.68 (1.80, 4.00)**	252 (50.3)	2.31 (1.51, 3.54)**	501
**Frequency of consuming SSB**							
Never	248 (69.7)	–	216 (60.7)	–	212 (59.6)	–	356
≤1 drink per week	406 (62.0)	–	369 (56.3)	–	277 (42.3)	–	655
2–3 drinks per week	134 (49.6)	–	133 (49.3)	–	76 (18.9)	–	270
Once per day	43 (31.9)	–	53 (39.3)	–	24 (17.8)	–	135
>1 times per day	31 (30.1)	–	35 (34.0)	–	19 (18.4)	–	103
**Frequency of thinking about an Australian SSB tax**							
Never	347 (43.3)	–	327 (40.8)	–	222 (27.7)	–	801
Once every 6–12 months	238 (69.2)	–	213 (61.9)	–	176 (51.2)	–	344
Once a month	161 (74.2)	–	152 (70.0)	–	118 (54.4)	–	217
Once a week	84 (71.8)	–	86 (73.5)	–	65 (55.6)	–	117
2–3 times per week	32 (80.0)	–	28 (70.0)	–	28 (70.0)	–	40

*p < 0.05, **p < 0.001; the control message block was used as the baseline to which all experimental blocks (2–4) were compared to, while the baseline for all other measures that was used in the logistic regression analyses is listed in the respective demographic. The *n* (%) values represent the somewhat- and strong-levels of support in each question for each subcategory.

^a^SEIFA: Socio-Economic Indexes for Areas—a suit of four indexes summarizing relative socio-economic advantages and disadvantages of geographic areas in Australia; higher scores represent socio-economic areas with greater advantage.

^b^The political party subgroup ‘Other’ includes Independent party groups/Unsure/Undecided individuals.

### Impact of message framing and support for an SSB tax in Australia

Overall, only small differences were observed in the responses to agreement-with, perceived effectiveness, and feeling-towards the SSB tax questions between those who received the different frames. When determining whether message framing increased support for an SSB tax the lowest level of agreement-with or positive feeling-towards the SSB tax was seen in those participants who received the control block (Table 4). Respondents who received Block 3 (in support of food industry) had 40% higher odds for feeling positive about the SSB tax compared to the control (OR = 1.40; 95% CI = 1.03, 1.90; *p* = 0.034). These results reflect the finding that positivity about the SSB tax was highest in the ‘support food industry’ frame (43.1%) and lowest with the control frame (35.3%).

**Table 4:** Distribution of respondents that were somewhat or strongly in support of the sugar-sweetened beverage tax (*n* (%)) stratified by message framing blocks and moderation analysis interaction results

	Agreement-with tax				Think tax will be effective				Positive feeling-towards tax			
	Block 1	Block 2	Block 3	Block 4	Block 1	Block 2	Block 3	Block 4	Block 1	Block 2	Block 3	Block 4
Political party	ns				ns				ns			
Greens	39 (76.5)	44 (81.5)	35 (72.9)	43 (72.9)	36 (70.6)	40 (74.1)	35 (72.9)	43 (72.9)	27 (52.9)	33 (61.1)	29 (60.4)	34 (57.6)
Labour	63 (51.2)	66 (56.9)	74 (55.6)	72 (55.8)	62 (50.4)	62 (53.5)	65 (48.9)	66 (51.2)	40 (32.5)	44 (37.9)	54 (40.6)	60 (46.5)
Other^a^	28 (49.1)	36 (42.4)	36 (47.4)	24 (43.6)	25 (43.9)	39 (45.9)	35 (46.1)	20 (36.4)	16 (28.1)	24 (28.2)	25 (32.9)	12 (21.8)
Liberal/National/Right-leaning	73 (52.3)	68 (52.3)	86 (64.7)	75 (56.4)	69 (50.4)	63 (48.5)	80 (60.2)	66 (49.6)	47 (34.3)	50 (38.5)	60 (45.1)	54 (40.6)
Geographical area	#				ns				ns			
Metropolitan	155 (60.1)	139 (53.1)	175 (62.7)	144 (56.7)	141 (54.7)	132 (50.4)	160 (57.4)	126 (49.6)	101 (39.2)	96 (36.6)	128 (45.9)	107 (42.1)
Rural	48 (45.3)	73 (61.3)*	53 (49.5)	67 (56.8)	51 (48.1)	69 (58.0)	51 (47.7)	67 (56.8)	29 (27.4)	54 (45.4)*	39 (36.5)	51 (43.2)
Age	ns				ns				ns			
18–24 years	26 (55.3)	46 (66.7)	22 (46.8)	30 (60.0)	25 (53.2)	48 (69.6)	26 (55.3)	31 (62.0)	15 (31.9)	32 (46.4)	11 (23.4)	19 (38.0)
25–40 years	82 (60.3)	71 (56.4)	81 (62.3)	64 (56.1)	81 (59.6)	63 (50.0)*	73 (56.2)	56 (49.1)	50 (36.8)	37 (29.4)	53 (40.8)	43 (37.7)
41–64 years	69 (53.1)	57 (46.7)	89 (59.7)	78 (53.8)	59 (45.4)	54 (44.3)	81 (54.4)	67 (46.2)	41 (31.5)	45 (36.9)	73 (49.0)	62 (42.8)
65 and over	26 (47.3)	40 (58.8)	39 (60.9)	42 (62.7)	27 (49.1)	39 (57.4)	35 (54.7)	41 (61.2)	24 (43.6)	37 (54.4)	31 (48.4)	36 (53.7)
Gender	ns				ns				ns			
Male	94 (51.1)	101 (52.6)	94 (55.3)	103 (54.2)	95 (51.6)	95 (49.5)	90 (52.9)	98 (51.6)	59 (32.1)	75 (39. 1)	73 (42.9)	76 (40.0)
Female	108 (59.3)	112 (58.3)	135 (62.5)	109 (59.2)	96 (52.8)	108 (56.3)	122 (56.5)	96 (52.2)	71 (39.0)	76 (39.6)	92 (42.6)	83 (45.1)

Block 1 = control; Block 2 = protecting teenagers; Block 3 = support food industry; Block 4 = anti-food-industry.

#Significant (*p* < 0.05) and, ns, non-significant difference found by the likelihood ratio test to compare original and interaction-term models;.

*p < 0.05, significant interaction was present in the subcategory.

Note: the ‘not sure’ category for geographical area was not presented in this table as there were no meaningful findings due to the small sample size.

^a^The political party subgroup ‘Other’ includes Independent party groups/Unsure/Undecided individuals.

While not statistically significant, the participants who received the ‘support food industry’ frame also had the highest levels of agreement-with, and belief that the tax will be effective (59.2% vs 55.2% in the control, and 55.1% vs 52.2% in the control, respectively).

The interaction results relevant to each hypothesis are presented in Table 4. Addressing the first hypothesis, left-leaning supporters who received the ‘anti-sugary drink companies’ frame did not show higher levels of support for the SSB tax, as no significant interaction was found. Notably, there was no significant interaction between the message frames and any political party preference. Although, when examining general trends, Greens party supporters were consistently most supportive of the SSB tax when receiving Block 2 (the ‘protecting teenagers’ frame). While conservative voters were consistently most in favour of the SSB tax when receiving Block 3, the ‘supporting food and drink companies’ frame (Table 4).

We also hypothesized that those living in rural areas were more likely to agree-with and have positive feelings to messaging which concerned protecting teenagers. The likelihood ratio test indicated that there was an interaction between the frame used and regionality (LR χ^2^(3) = 9.43; *p* = 0.0241). Specifically, respondents from rural areas were significantly more likely to agree-with the SSB tax when they received the message frame regarding ‘protecting teenagers’ (Block 2) (61.3% for Block 2 vs 45.3% for the control block (*p* = 0.009)). While the overall likelihood ratio test when assessing positive feelings and the interaction between frame and regionality was not statistically significant (*p* = 0.0597), those from rural areas who received the ‘protecting teenagers’ frame (Block 2) had significantly more positive feelings towards the SSB tax (45.4% in Block 2 vs 27.4% in control, *p* = 0.019) (Table 4).

No evidence was found to support our third hypothesis, that older individuals who received the pro-food-industry frame would have greater support for the SSB tax. Gender also did not have a significant effect on support for the SSB tax when receiving different message frames. Thus, we found no evidence for our fourth hypothesis that females were more likely to be swayed by messaging focused on protecting children and teenagers. Although, for males, support for the SSB tax was highest when they received the ‘supporting food and drink companies’ frame.

All other interaction terms were non-significant (*p* > 0.05).

## DISCUSSION

Using an experimental framing study, we sought to understand whether political party voting preference and sociodemographic factors affect individuals’ support for an SSB tax in Australia, and whether message framing affects this support. When considering solely message framing, we found that there was little difference between the support-for, and perceived effectiveness of an SSB tax between groups who received different frames. However, positive feelings towards the tax were highest in the group who received the ‘supportive of food and drink companies frame’. Overall participants who received the control message were least supportive of the SSB tax, but the difference in support was marginal.

We also explored the impact of political affiliation and sociodemographic factors on support for an SSB tax, and specifically, the interaction of these factors with message frames. We had expected that framing our message to appeal to the values and interests of the Australian public would increase support for an SSB tax. However, when we introduced message frames, support for the SSB tax was only significantly higher for those living in rural areas who received the ‘protecting teenagers’ frame. We found no evidence to support our other hypotheses pertaining to political affiliation, age and gender. The limited impact of the frames was surprising as the messages had been developed to align with the theory of concept accessibility by appealing to pre-existing beliefs and values of Australians identified in a previous study (Cullerton *et al.*, 2022). The theory of concept accessibility and previous research has found that frames are most effective when they appeal to pre-existing interests, or political or other cultural values of an audience (Cacioppo, 1986; Nisbet, 2009). The limited impact of the message frames in our study could be due to a number of reasons.

Firstly, the sample used in the original qualitative interviews which guided the message development may not have been large or varied enough to capture the key interests and values around SSB taxes for the Australian population. Other framing studies have identified that ensuring personal relevance of a message is needed to ensure engagement (Marquina *et al.*, 2022). While we attempted to appeal to personal interests, it is possible that our messages were too general and only peripherally engaged the audience, resulting in messages having less effect. Also, in line with framing research, we only manipulated a small amount of text resulting in small differences between the messages. This limited differentiation may have impacted on the level of meaningful engagement.

Secondly, previous studies have found that personal characteristics of respondents, not the type of message, are better predictors of policy support (Kormos and Gifford, 2014; Whitley *et al.*, 2018; Marquina *et al.*, 2022). This finding corresponds to our results where political affiliation with the Greens party, having a university education, watching the news frequently and consuming low levels of SSBs were consistently associated with higher levels of support towards an SSB tax regardless of the frame participants received. When considering these findings it is logical to conclude that those who engage less in the targeted behaviour or who are more aware of the harms associated with certain behaviours, would be more likely to find policies acceptable (Diepeveen *et al.*, 2013). Conversely, those who engage more in the targeted behaviour will be less likely to support a policy that aims to decrease that behaviour (Diepeveen *et al.*, 2013). Therefore in some cases people may have formed a position about SSB taxes rooted in their values, beliefs and norms which will be very hard to change with a simple rhetorical frame (Gollust *et al.*, 2017).

In support of our hypothesis, we found that those living in rural areas were more likely to be receptive to messaging which concerns protecting teenagers. This may relate to the importance of family values in rural areas (Heenan, 2010). This finding has important implications for future advocacy efforts as it highlights the importance of nuance in messaging. While it is extremely efficient for advocates to use one message to target a population, our findings highlight that targeted messages for different population groups may be required. Further, the rural population of Australia are more likely to be conservative voters and understanding messaging that resonates with this group is particularly beneficial for advocates as conservative voters may be less inclined to support fiscal policies.

Another important finding was the trend that respondents who received the ‘supportive of food and drink companies’ frame generally had higher levels of support for the SSB tax and felt more positive about it than those receiving the control frame. Similarly, males who received this frame were more supportive as were conservative voters. This finding corresponds to previous research we conducted where participants expressed concern about the impact of taxes on the SSB industry and did not want to disadvantage them (Cullerton *et al.*, 2022). As public health advocates, there is a tendency to demonize the ultra-processed food industry; however, this approach may backfire as it can alienate the public and decrease their level of support towards the policy (as we saw with the anti-sugary drink companies frame). Even though there may be concern regarding the practices of the food industry it is important for advocates to remember what messages may resonate with the public when communicating about public health policy. Alternatively, if advocates wish to adopt this messaging approach, they may wish to take on board lessons from other framing studies which have shown that support for an SSB tax can be increased by proposing positive uses for funds raised by extra taxation even while criticizing harmful industry sectors (Koon and Marten, 2023).

The lack of association between being a left-leaning party voter and positively responding to the anti-sugary drink companies frame was surprising as according to values-based framing theory (Chong and Druckman, 2007) this should align with progressive values. However, we can gain further insight into this result when we examine general levels of support for the tax by political party (without message frames). Greens voters had very high levels of support for the SSB tax and so the lack of movement associated with message frames may be due to the ceiling effect, that is, when the majority of participants already agree-with a proposed policy, limited responsiveness to framing will occur. This effect has been seen in climate change framing experiments with left-leaning voters showing limited response to different frames (Feldman and Hart, 2018).

Interestingly, participants who voted for conservative parties and for Labour (a centre-left party) had similar levels of agreement across all three measures towards an SSB tax, which was considerably lower than participants who voted for the Greens. While the conservative voters’ stance aligns with conservative ideology, that is, supporting limited market interference, the result from Labour party voters was surprising as they are considered a left-leaning party (Fenna and Manwaring, 2021). However, while Labour voters may be broadly progressive regarding certain economic and cultural issues, this may not translate to public health issues (Taylor, 2019). This is an area that needs further investigation.

The undecided or swing voters generally had the lowest levels of support for the SSB tax and the different frames had limited impact on them. This finding could relate to the importance of personal characteristics that may impact on beliefs towards the policy, for example, low level of education, minimal interest in news or distrust of the government. Undecided voters are an important group for politicians as they can make or break elections (Cullerton *et al.*, 2021) and this finding may explain why an SSB tax has received no interest from Australian politicians from mainstream political parties. Changing the beliefs of this group may be a particular challenge for advocates but remains critical for an SSB tax to proceed.

### Strengths and limitations

This study used quotas representative of the Australian population by gender, age, education and regionality. We also collected data on political party voting behaviour which adds new insights into this topic. However, there are important limitations associated with this study. While the use of a population-based sample extends the study’s external validity, the artificial setting of an internet-based survey is considerably different to the real-life contexts in which people are exposed to or select information about policy issues within a competitive media environment (Gollust *et al.*, 2013). In real-world settings, individuals are often exposed to multiple frames simultaneously, with the source of the message also playing a crucial role in how persuasive a message frame is (Chong and Druckman, 2007). Our study did not examine these multifaceted dimensions of messaging. Further, using an online panel company and our own social media recruiting strategy may have limited the coverage and selection of participants. Also, to avoid priming we chose not to collect baseline measurements of policy support. Consequently, we could not examine whether participants’ prior policy attitudes moderated the magnitude or direction of the message effects. Finally, given the number of interactions that were investigated, there is a risk that a Type I error (false positive) may have occurred. In order to decrease this risk we ensured that the interaction hypotheses were pre-defined based on the literature.

## CONCLUSION

We examined whether political party voting preference and sociodemographic factors affect individuals’ support for an SSB tax, and whether message framing affects this support. We found no clear effect of message framing with the association between the frames used and SSB support limited. However, positive feelings towards an SSB tax were highest in the group that received the ‘support food and drink companies’ frame, while participants who received the control frame recorded a marginally lower level of support and positivity towards the SSB tax.

Studying the influence of the frames among different sub-groups few clear associations were found except for the ‘protecting teenagers’ frame resulted in higher levels of support from rural participants. Regardless of frames used, we saw higher levels of support for an SSB tax among Greens voters, those with higher levels of education, those who watch the news frequently and those who consume minimal quantities of SSB drinks. Our findings demonstrate that using persuasive language that resonates with participants may only have a small impact overall and that support for policies such as the SSB tax varies more based on the personal characteristics of participants.

The present study adds to our understanding of public opinion in the context of public health policies and the role framing plays. More research is needed to understand the contextual elements of framing and how it impacts different population groups, for example rural versus urban voters. We also need more research examining undecided or swing voters and their beliefs around public health policy. This is a crucial group for advocates to target and understanding as much as possible about their beliefs and values will only help further advocacy efforts.

## SUPPLEMENTARY MATERIAL

Supplementary material is available at *Health Promotion International* online.

daad193_suppl_Supplementary_Appendixs_1
