# Supplementary material for: Effect of message framing on support for a sugar-sweetened beverage tax in Australia: a cross-sectional survey analysis
Source: Health Promot Int. 2024 Jan 11;39(1):daad193. doi: 10.1093/heapro/daad193 (PMC10783238; doi:10.1093/heapro/daad193)
Supplement: daad193_suppl_Supplementary_Appendixs_1 [file daad193_suppl_supplementary_appendixs_1.docx]

**SUPPLEMENTARY FILES**

Appendix 1: description of survey and response options

| Concept | Survey question prompt | Response options | Use in logistic regression and overall analysis |
| --- | --- | --- | --- |
| Age | Are you 18 years or over? (Q27) | Yes | Screening for survey participation eligibility; included. |
|  |  | No | Excluded |
| Involvement with the food sector | Do you work in the manufactured food sector or as a nutrition professional? (Q28) | Yes | Screening for survey participation eligibility; included. |
|  |  | No | Excluded |
| Citizenship status | Are you an Australian citizen? (Q29) | Yes | Screening for survey participation eligibility; included |
|  |  | No | Excluded |
|  | Are you the main food shopper in your household? (Q10) | Yes |  |
|  |  | No |  |
|  |  | Shared |  |
| Agreement for SSB | How much do you agree with the introduction of a 20% tax on sugary drinks? (Q11) | Somewhat agree, strongly agree | Agreed (1) |
|  |  | Neither agree nor disagree, somewhat disagree, strongly disagree | Not agree (0) |
| Perceived effectiveness of SSB | How *effective* do you think a 20% sugary drinks tax would be in improving the health of Australians? (Q12) | Very effective, somewhat effective | Effective |
|  |  | Neutral, not effective, really not effective | Not effective |
| Feelings toward SSB | How do you *feel* about a 20% sugary drinks tax? (Q13) |  |  |
|  |  |  |  |
